# Dried blood spots PCR assays to screen congenital cytomegalovirus infection: a meta-analysis

**DOI:** 10.1186/s12985-015-0281-9

**Published:** 2015-04-14

**Authors:** Li Wang, Xiaoxing Xu, Huiping Zhang, Jihong Qian, Jianxing Zhu

**Affiliations:** Department of Neonatology, Xinhua Hospital, Shanghai Jiaotong University School of Medicine, 1665 Kongjiang Road, Shanghai, 200092 China; Department of Clinical Epidemiology, Xinhua Hospital, Shanghai Jiaotong University School of Medicine, 1665 Kongjiang Road, Shanghai, 200092 China

**Keywords:** Congenital cytomegalovirus infection, Dried blood spots, Neonates, Screen

## Abstract

The performance of dried blood spots (DBS) polymerase chain reaction (PCR) assays in screening for congenital cytomegalovirus (cCMV) infection varies between different studies. To determine whether the DBS PCR assay has sufficient accuracy to be used as a screening test for cCMV infection, we performed a meta-analysis of 15 studies (n = 26007 neonates) that evaluated the performance of DBS PCR tests in screening for cCMV infection and that met our inclusion criteria. The pooled sensitivity and specificity were 0.844 (95% CI = 0.812–0.872) and 0.999 (95% CI = 0.998–0.999), respectively, and the diagnostic odds ratio was 1362.10 (95%CI = 566.91–3272.60). As sensitivity analysis showed that the results were robust. In conclusion, the performance of DBS PCR assays for testing cCMV was more suitable for retrospective diagnosis than screening.

## Background

Cytomegalovirus (CMV) causes developmental defects at birth among 10% of infected babies, and 8.5–18% of asymptomatic newborns will develop sensorineural hearing loss (SNHL) [1,2]. The two main potential benefits of neonatal screening are early intervention to prevent the onset or progression of SNHL and the identification of infants at risk for late-onset or progressive SNHL [3]. Randomized control trials (RCT) and observational studies have reported that ganciclovir therapy begun in the neonatal period in symptomatically infected infants prevented hearing deterioration at 6 months [4-6].

The gold standard for the diagnosis of congenital CMV (cCMV) infection is positive results for viral isolation from urine and/or saliva collected during the first 3 weeks of life [7]. This method is not suitable for large scale screening. As an alternative, dried blood spots (DBS) polymerase chain reaction (PCR) assays are getting more and more attention, because specimens can be collected routinely and preserved easily; and because PCR can be automated. Certain researchers have suggested that this method is suitable for the retrospective diagnosis of cCMV infection in infants or children with hearing loss, mental retardation or other symptoms compatible with cCMV [8]. However, others have insisted the tests is not suitable for screening due to low sensitivity. In conclusion, there is no consensus on the screening performance of DBS PCR assay in screening for cCMV infection.

In the present meta-analysis, we systematically reviewed studies of the diagnostic performance of DBS PCR assays for cCMV infection to determine whether these assays are sufficiently effective to be used for screening neonates.

## Methods

### Search strategy

Shibata et al. [9] first reported a method for using DBS PCR to detect CMV DNA in 1994, so the publication time was limited from 1990 to 2014. We performed an electronic search of Medline (1990 to January 31, 2014), the Cochrane Library database (1990 to January 31, 2014), and the Science Citation Index (1990 to January 31, 2014) using the following search terms: “DBS” (or “dried blood spots”, “filter papers” or “Guthrie card”), and “congenital cytomegalovirus”.

### Study eligibility

The inclusion criteria were as follows:

1. Studies that compared DBS PCR assays with the standard method for detecting cCMV infection. The DBS samples had to have been collected within the first week of life. The protocol for DBS PCR assays included DNA extraction from DBS samples and PCR amplification of CMV DNA. Reference standards were generally viral isolation from or PCR detection in urine and/or saliva collected within the first 3 weeks of life.
2. Studies with available data for constructing contingency tables for true positive (TP), false positive (FP), false negative (FN) and true negative (TN) determination.

The exclusion criteria were as follows:

1. Studies that did not compare DBS PCR testing with standard tests (viral isolation from urine and/or saliva) for diagnosing cCMV infection.
2. Studies that overlapped with the studies selected (i.e., studies from the same study group, institution, and period of inclusion).
3. Letters, editorials, expert opinions, and reviews without original data, and case reports.

### Data extraction

A comprehensive search of the literature was performed by two of our authors, who each assessed which studies to retain based on the inclusion and exclusion criteria. Each investigator was blinded to the other’s selections. Discrepancies between the two were arbitrated by our senior investigator (Jihong Qian). All data were recorded, regarding the study setting, screening test properties and screening test results, such as TPs, FPs, FNs, and TNs. When several groups of CMV DNA tests were examined in one study, the data from the highest sensitivity group were chosen for analysis.

### Limit of detection (LOD) calculation

8 of 14 including studies reported the protocols of DNA extraction and PCR, and the detection limit of PCR reaction (copies/1 ml whole blood or copies/1 reaction). We extracted data from 5 of the 8 studies directly [10-14], and referenced the handbook of PCR kit used in 3 of the 8 studies [2,15,16] without exact protocol. We calculated the LOD (copies/1 ml whole blood) by the method shown in Table 1, based on assuming a yield of 100% extraction.

**Table 1:** **The calculation methods of LOD**

**Author,Year**	**Blood sample volume V1**	**Dilution volume V2**	**Elute used in PCR assay volume V3**	**Whole blood volume in each PCR reaction V4**	**LOD1 copies/PCR reaction**	**LOD2 copies/ml whole blood**
Barbi et al [10]	50	25	2	4	2	500
Binda et al [11]	40	45	10	8.89	4	449.9
Boppana et al [2]	8	30^a^	5	1.3	1.56	1200^d^
Leruez-Ville et al [12]	50	50	10	10	40	4000
Paradizˇ et al [13]	16	100	20	3.2	4.15	1296
Scanga et al [14]	50	100	10	5	8	1600
Soetens et al [16]	50	25	5^c^	10	94	9400
Vaudry et al [15]	50	50^b^	5	5	8	1600

**a.** The volume is recommended in the DNA extraction kit (Qiagen) used in the study, may not the true volume in the tests. **b.** The volume is recommended in the DNA extraction kit (Magazorb, USA) used in the study, may not the true volume in the tests. **c.** The data was referenced study by Leruez-Ville et al [36]. **d.** We chose the minimum dilution in the sensitivity titration assays to evaluate the LOD, because the data described in the article seems not reliability.

$\mathrm{L}\mathrm{O}\mathrm{D}1=\frac{LOD2\times {\mathrm{V}}_4}{1000}=\frac{LOD2\times {V}_3\times {V}_1}{1000\times {V}_2}$ (Assuming a 100% yield of extraction).

### Qualitative assessment

Evidence quality was assessed by two independent investigators using the Quality Assessment of Diagnostic Accuracy Studies 2 (QUADAS-2) tool [17]. Briefly, the QUADAS-2 includes 4 key domains that are rated in terms of the risk of bias: patient selection, execution of the index test, reference standard, and flow of patients (in particular, whether there was an appropriate interval between the index test and the reference standard). Each domain is scored as having a high, low, or unclear risk of bias. Discrepancies regarding the risk of bias and other discrepancies were arbitrated by a third reviewer.

### Test for heterogeneity

The values of *I*^*2*^ and Cochrane -Q were assessed for heterogeneity among studies. *I*^*2*^ values of 25%, 50% and 75% represent mild, moderate, and severe inconsistency, respectively.

### Sensitivity analysis

In the sensitivity analysis, one study at a time was excluded from each analysis. Most of the results appeared to be robust to the influence of individual studies (Table 2). The diagnostic odds ratio (DOR) results also did not vary significantly when the reference changed.

**Table 2:** **Sensitivity analysis**

**The influence of each trial for the outcome of the meta-analysis**		
**First Author (Year)**	**DOR**	**95%CI**
Barbi et al [10]	1350.5	532.0-3428.5
Barbi et al [8]	1090.5	453.4-2623.0
Binda et al [11]	1203.6	502.3-2884.3
Boppana et al [2]	1396.4	501.6-3887.2
Boppana et al [2]	1212.7	482.7-3046.9
Distéfano et al [24]	1275.9	509.2-3197.1
Johansson et al [22]	1566.9	651.6-3768.1
Leruez-Ville et al [12]	1287.4	508.7-3258.3
Leruez-Ville et al [26]	1243.8	495.8-3120.4
Paixão et al [25]	1682.7	691.6-4094.1
Paradizˇ et al [13]	1262.6	508.9-3132.5
Scanga et al [14]	1432.5	576.3-3561.0
Soetens et al [16]	1636.5	711.2-3765.3
Vaudry et al [15]	1555.8	642.7-3765.9
Yamamoto et al [20]	1357.9	536.4-3437.6
combined	1262.6	508.9-3132.5

The results showed that the diagnostic odds ratio (DOR) results did not vary significantly when the reference changed.

### Statistical methods

The statistical analysis was performed using Meta-Disc (version 1.4) and STATA 12.0 software. The pooled sensitivity, pooled specificity, positive likelihood ratio (LR+), negative likelihood ratio (LR-), positive predictive values (PPVs), negative predictive values (NPVs) and their 95% CIs were calculated using the random effect model and the fixed effect model [18,19]. The joint distribution of the TP rate (TPR) and FP rate (FPR) was analyzed using a summary receiver operating characteristic (SROC) curve. The performance of the DBS PCR assays was analyzed based on the DOR, the Q-statistic, and the area under the ROC curve (AUC) [20]. Heterogeneity among studies was analyzed using the *I*^*2*^ statistic.

## Results

### Eligible studies

**Figure 1:**
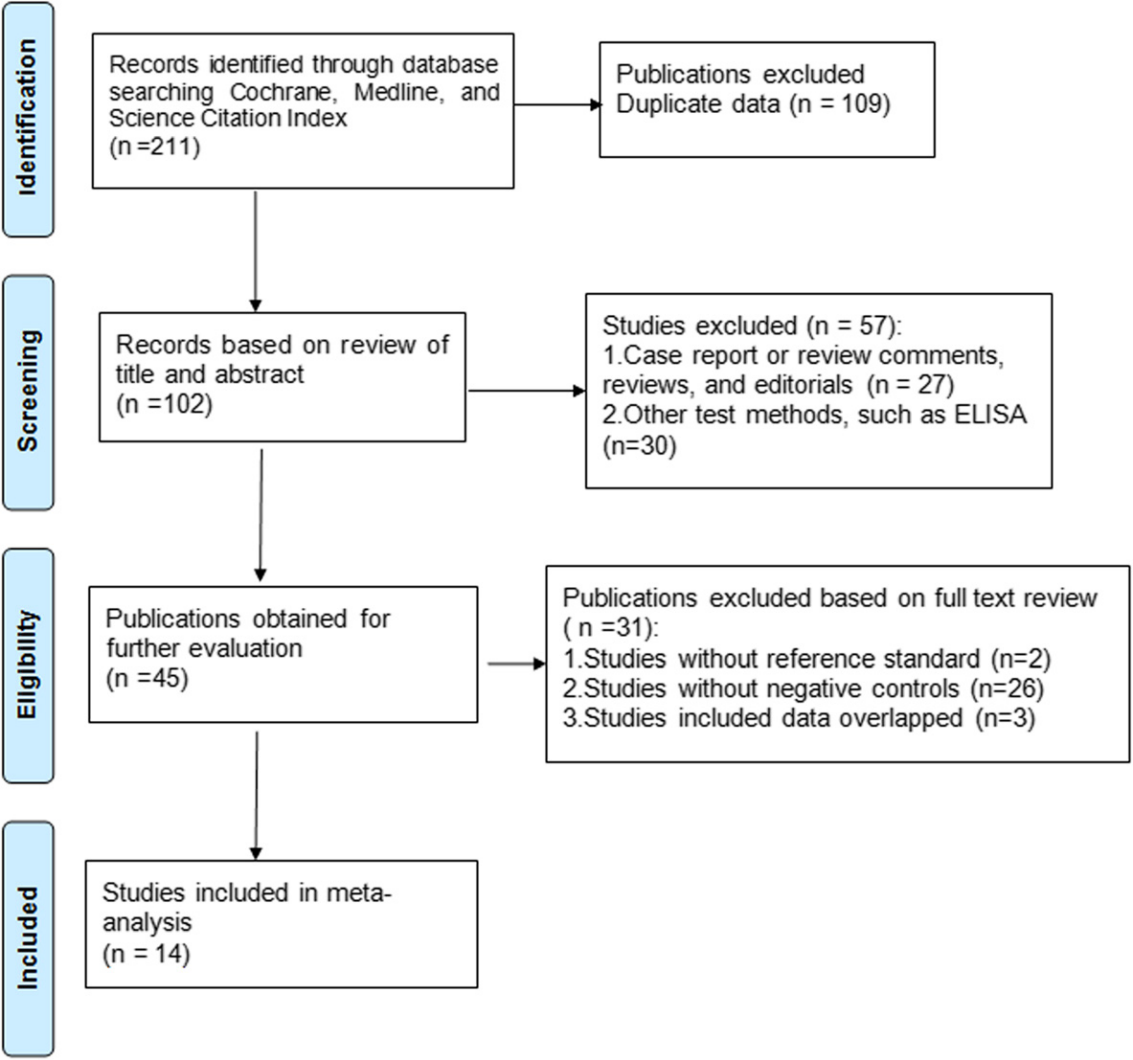
Flow chart showing the process of study selection. 14 articles were included in this meta-analysis.

The searches for screening generated 102 articles after removal of duplicates. Through assessment for eligibility, 14 articles were included in the meta-analysis (details in Figure 1). In 2010, Boppana et al. [2] enrolled two groups of subjects, who were screened for cCMV using two different CMV DNA tests (single-primer real-time PCR and two-primer real-time PCR). This article was deemed to be two independent studies, and thus 15 studies were included in the present review. The main characteristics and details of the studies are reported in Table 3. Overall, 26007 neonates were enrolled, composed of 583 neonates identified as having cCMV infection and 25424 neonates without CMV infection, as determined using a reference standard.

**Table 3:** **Characteristics of the included studies**

**Author [Reference]**	**Year**	**Location**	**Design**	**Total population screened**	**Inclusion criteria**	**Size of DBS used (diameter)**	**DBS stored temperature**	**Time of samples tested to collected**	**Type of DNA extraction**	**Type of PCR**	**Target region**	**Gold standard**
Barbi et al [10]	1998	Italy	Prospective	205	Universal screening	3 disks (3mm)	UN	UN	Heat shock	Nested-PCR	UL55	Viral isolation from saliva
Barbi et al [8]	2006	Italy	Retrospective	874	Suspected of cCMV infection	3 disks (3mm)	UN	UN	Heat shock	Real-time PCR	gB	Viral isolation from urine or saliva
Binda et al [11]	2004	Italy	Retrospective	195	Suspected of cCMV infection	3 disks (3mm)	UN	UN	modified Heat shock	Nested-PCR	gp58	Viral isolation from urine,saliva or blood
Boppana et al [2]	2010	America	Prospective	11407	Universal screening	2 disks (3mm)	room temperature	14.6±9.6days	Qiagen M48 robotic system	Single-primer Real-time PCR	gB	The DEAFF assay on the follow-up saliva/urine sample
Boppana et al [2]	2010	America	Prospective	9018	Universal screening	2 disks (3mm)	room temperature	14.6±9.6days	Qiagen M48 robotic system	Two-primer Real-time PCR	gB & IE2	The DEAFF assay on the follow-up saliva/urine sample
Distéfano et al [24]	2008	Argentina	Retrospective	145	Compatible symptoms	UN	UN	UN	Heat shock	Nested-PCR	gB	Viral isolation from urine
Johansson et al [22]	1997	Sweden	Retrospective	31	infants confirmed with/without cCMV and infants whose samples were stored close to the infetive ones	25mm2	4°C	12-18 years	Phenol-chloroform	PCR+hybridization test	OP1, OP2, IE1	Viral isolation from urine
Leruez-Ville et al [12]	2009	France	Retrospective	214	Compatible symptoms, Maternal PI	UN	UN	UN	QiAamp DNA Blood Mini kit (Qiagen)	Real-time PCR	UL123exon 4	Urine culture/PCR
Leruez-Ville et al [26]	2011	France	Prospective	271	Compatible symptoms, Maternal PI	whole spot (10mm)	standard conditions	After metabolic screening	QiAamp DNA Blood Mini kit (Qiagen)	Real-time PCR	UL123exon 4	Urine sample PCR
Paixão et al [25]	2009	Portuguese	Retrospective	308	Neonates confirmed with/without cCMV infection	UN	UN	UN	Heat shock	Nested-PCR	gp58	Urine shell-viral culture
Paradizˇ et al [13]	2012	Slovenia	Prospective	2841	Universal screening	8 discs (2mm)	room temperature	7 days	QiAamp DNA Blood Micro kit (Qiagen)	Real-time PCR	UN	Urine sample PCR
Scanga et al [14]	2006	America	Retrospective	19	infants confirmed with/without cCMV and infants whose samples were stored close to the infetive ones	whole spot (10mm)	room temperature	2-20 months	QiAamp DNA Blood Micro kit (Qiagen)	Real-time PCR	POL	Urine culture
Soetens et al [16]	2008	Brussels	Retrospective	67	Universal screening	whole spot (10mm)	room temperature	72.9±31 months (0.5-130 months)	Phenol chloroform	Conventional PCR + nested-PCR	US8 & gH	Urine culture
Vaudry et al [15]	2010	Canada	Prospective	95	infants with VLBWs or SGA	UN	UN	about one month	MagaZorb DNA extraction Kit (Cortex Biochem)	CMV LC-real time PCR (Roche Diagnostic)	gB1, gB2	Viral isolation from throat swabs
Yamamoto et al [21]	2001	Brazil	Prospective	332	Universal screening	3 disks (6mm)	-20°C	UN	Heat shock	Nested-PCR	MIE & gB/gB1 & IE	Urine sample viral culture and/or PCR

We extracted characteristics of the 15 studies, regarding to sample group information, DBS sample collection, DNA extraction and PCR methods. In Boppana et al. [2] study, they enrolled two groups of subjects and used different methods, therefore this article was deemed to be two independent studies. In Distéfano et al [24] study, 15 of 145 cases were identified as perinatal CMV infection, they were not included in this meta-analysis.

### Quality assessment

The quality of the eligible studies was assessed according to the QUADAS-2 criteria (see in Table 4). Four of the 15 studies were considered to having a low risk of bias in any of the domains [2,15,21]. Nine studies were assessed as having a high risk of patient selection bias, because the neonates with classical symptoms of cCMV infection, confirmed using reference standard might have been more easily diagnosed with cCMV [10-12,14,22-26]. Seven studies (5 with high risks and 2 with unclear risks) were identified as high risks in the index test domains [12-14,16,22,23,25]. In these studies, the researchers knew the results of the reference standard, which could have influenced the interpretation of the index test results. One study [11] had an applicability concern in the index test domains, because the study employed two PCR methods, conventional nested PCR designed to amplify one region in the gp58 gene and a commercial kit for the amplification of the IE gene. In 3 studies [11,12,23], there was several unclear risks because the study had not been published as open access or because the article did not describe related information, which were considered to be potential risks.

**Table 4:** **Summary of the assessment of the included studies using QUADAS-2**

**QUADAS-2 quality assessment**							
**Study (Year)**	**Risk of bias**			**Applicability concerns**			
	**Patient selection**	**Index test**	**Reference standard**	**Flow and timing**	**Patient selection**	**Index test**	**Reference standard**
Barbi et al [10]	H	L	L	L	L	L	L
Barbi et al [8]	H	?	L	L	?	?	L
Binda et al [11]	H	L	?	L	L	H	L
Boppana et al [2]	L	L	L	L	L	L	L
Boppana et al [2]	L	L	L	L	L	L	L
Distéfano et al [24]	H	L	L	L	L	L	L
Johansson et al [22]	H	H	L	L	L	L	L
Leruez-Ville et al [12]	H	?	L	L	L	L	L
Leruez-Ville et al [26]	H	L	L	L	L	L	L
Paixão et al [25]	H	H	L	L	L	L	L
Paradizˇ et al [13]	L	H	L	L	L	L	L
Scanga et al [14]	H	H	L	L	L	L	L
Soetens et al [16]	L	H	L	L	L	L	L
Vaudry et al [15]	L	L	L	L	L	L	L
Yamamoto et al [20]	L	L	L	L	L	L	L

H = High risks; L= Low risks; ? = Unclear risks.

### Test for heterogeneity

The threshold effect was denied by diagnostic threshold analysis (Spearman correlation = 0.415, P = 0.124). The *I*^*2*^ statistics was 45.3% and the Cochrane-Q statistics was 25.57 (P = 0.0293) for the pooled DOR, suggesting that there was moderate heterogeneity among the included studies.

### Screening performance

**Figure 2:**
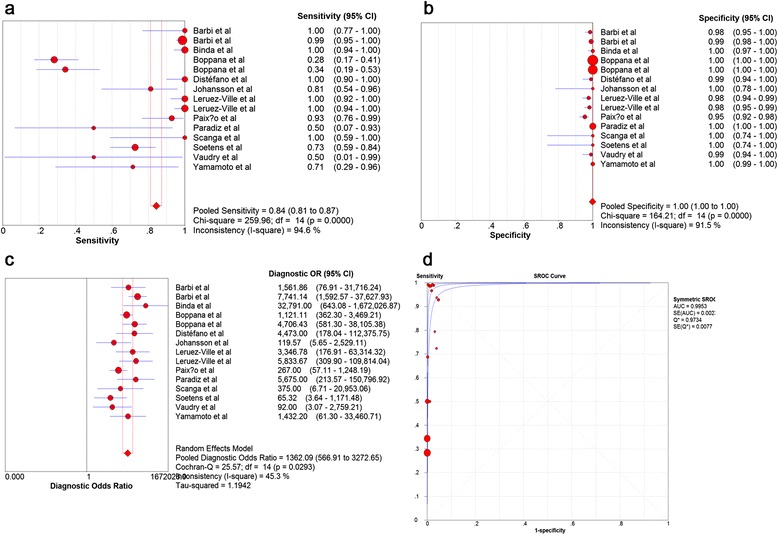
Summary of diagnostic performance estimates using random effect model. **a**. Pooled sensitivity of DBS PCR assays; The pooled sensitivity is 0.84. **b**. Pooled specificity of DBS PCR assays; The pooled specificity is 1.00. **c**. DOR for DBS PCR assays; The DOR is 1362.09. **d** SROC curve for cCMV diagnosis by DBS PCR assays. The AUC is 0.9953.

A total of 15 studies including 26007 DBS samples from neonates were assessed. The pooled sensitivity and specificity of the DBS tests were 0.844 (95% CI = 0.812–0.872) and 0.999 (95% CI = 0.998–0.999), respectively (Figure 2a, b). The pooled LR+ was 99.437 (95% CI = 45.666–216.523), and the pooled LR- was 0.110 (95% CI = 0.0424–0.289). The pooled PPV and NPV were 0.906 (95% CI = 0.835–0.948) and 0.991 (95%CI = 0.972–0.997), respectively. The pooled DOR was 1362.10 (95% CI = 566.91–3272.60) using the random effect model (Figure 2c). The AUC was 0.9953 (SE = 0.0023) with Q* = 0.9734 (SE = 0.0077) (Figure 2d), showing that the DBS tests for cCMV performed well. The pooled data were analyzed using fixed effect model, and the results were consistent with those of the analysis using the random effect model (data not shown).

### Subgroup analysis and meta regression

A subgroup analysis of the study design (Table 5a) was performed because the 7 prospective studies [2,10,13,15,21,26] had lower sensitivity than the retrospective studies [11,12,14,16,22-25]. The results showed that the 7 prospective studies, with no significant heterogeneity had obviously lower sensitivity (0.623) than the retrospective studies (0.945). However, the diagnostic performance in the two subgroups of studies was similar, as evidenced by the DOR and the AUC. In addition, there was moderate heterogeneity in subgroup of retrospective, indicated there was other factors to affect the heterogeneity.

**Table 5:** **Performance of subgroups of DBS PCR assays for screening cCMV**

**a. Subgroup analysis of study design and diagnostic accuracy**								
**Study Design**	**N**	***I2(%)***	***P***	**Pooled sensitivity (95% CI)**	**Pooled specificity (95% CI)**	**Pooled LR+ (95%CI)**	**Pooled LR- (95%CI)**	**DOR (95%CI)**
Prospective	7	0.0	0.45	0.623 (0.548 - 0.693)	0.999 (0.999 - 1.000)	280.72 (60.026 - 1312.8)	0.374 (0.182 - 0.768)	1573.9 (699.17 - 3543.00)
Retrospective	8	64.0	0.01	0.945 (0.918 - 0.965)	0.983 (0.974 - 0.989)	43.831 (19.745 - 97.298)	0.043 (0.007 - 0.280)	1085.71 (229.94 - 5126.46)
**b. Subgroup analysis of LOD and diagnostic accuracy**								
**LOD**	**N**	***I2(%)***	***P***	**Pooled sensitivity (95% CI)**	**Pooled specificity (95% CI)**	**Pooled LR+ (95%CI)**	**Pooled LR- (95%CI)**	**DOR (95%CI)**
High group	4	27.5	0.25	0.853 (0.773 - 0.914)	0.983 (0.960 - 0.994)	34.910 (16.013 - 76.015)	0.118 (0.009 - 1.596)	302.47 (44.653 - 2048.9)
Low group	5	19.4	0.29	0.612 (0.534 - 0.658)	1.000 (0.999 - 1.000)	554.64 (91.139 - 3375.4)	0.378 (0.166 - 0.862)	2428.5 (795.47 - 7413.9)
Others	6	61.1	0.0249	0.970 (0.945 - 0.986)	0.985 (0.978 - 0.990)	53.316 (21.532 - 132.013)	0.053 (0.011- 0.260)	1438.9 (300.50 - 6890.40)
**c. Subgroup analysis of area of DBS and diagnostic accuracy**								
**Diameter**	**N**	***I2(%)***	***P***	**Pooled sensitivity (95% CI)**	**Pooled specificity (95% CI)**	**Pooled LR+ (95%CI)**	**Pooled LR- (95%CI)**	**DOR (95%CI)**
Large	5	44.3	0.13	0.861 (0.792 - 0.914)	0.999 (0.997 - 1.000)	103.91 (18.832 - 573.350)	0.116 (0.038 - 0.729)	1041.8 (151.62 - 7159.1)
Small	5	41.0	0.15	0.632 (0.557 - 0.702)	1.000 (0.999 - 1.000)	260.02 (40.310 - 1677.3)	0.295 (0.122 - 0.711)	1656.0 (421.89 - 6499.8)
Others	5	64	0.01	0.945 (0.918 - 0.965)	0.983 (0.974 - 0.989)	43.831 (19.745 - 97.298)	0.043 (0.007 - 0.280)	1085.71 (229.94 - 5126.46)

**a.** Diagnostic performance of DBS PCR assays in study design subgroups. The sensitivity in retrospective studies was higher than that in prospective studies, 94.5% and 62.3% respectively. *I* ^*2*^ (64.0%) in subgroup of retrospective studies indicated that there was moderate heterogeneity. **b.** Diagnostic performance of DBS PCR assays in LOD subgroups. The test performance in low-LOD group was better than that in another two groups. The DOR were 2428.50 and 302.47 respectively. But the sensitivity in low-LOD subgroup (61.2%) was lower than that in high-LOD group (85.3%). **c.** Diagnostic performance of DBS PCR assays in area of DBS subgroups. The sensitivity of test in large area subgroup was better than that in small area subgroup, 86.1% and 63.2% respectively.

The limit of detection (LOD) of DBS assays is critical to sensitivity. We extracted and obtained LOD data from 9 studies [2,10-16,22], whereas the remaining 6 studies had no LOD data. A subgroup analysis of the LOD was performed among a high-LOD group (LOD ≥ 1500 copies/ml) [12,14-16], a low-LOD group (LOD < 1500 copies/ml) [2,10,11,13] and others (the group of remaining studies without LOD data) [23-27]. The sensitivity in the high-LOD group was higher than that in the other two groups, so it is possible that low LOD led to a higher false positive rate (Table 5b).

The DBS samples were all not uniform, with different sample sizes, storage temperatures and areas of the samples tested. We chose to analyze the most important factor that may influence the sensitivity: the total surface area of DBS. Three groups, large area group (>25 mm^2^) [13,14,16,21,26], small area group (≤25 mm^2^) [2,10,11,22,23], and others (the group of remaining studies with unknown the area data) [12,15,24,25], were compared by subgroup analysis. The results indicated that the large surface area group had higher sensitivity (Table 5c).

We performed meta-regression (Table 6) to investigate whether the above factors influenced diagnostic performance. The LOD, PCR methods and area of DBS sample were the factors analyzed in the meta-regression. The results showed that only the PCR method was related to the performance. The number of included studies were not enough to perform meta-regression of 4 factors, therefore we exclude the “study design” factor. If the factor “study design” was added into the meta-regression, the results were not changed.

**Table 6:** **Meta-regression to determine potential sources of heterogeneity**

**Possible sources of heterogeneity of meta -analysis**				
**study characteristic**	**Coefficient**	***P value***	**Relative DOR**	**95% CI**
LOD	0.33	0.5304	1.39	0.45 - 4.34
Area	-0.14	0.7802	0.87	0.28 - 2.65
PCR (real-time PCR vs.nested-PCR vs. others)	-1.68	0.0082	0.19	0.06 - 0.58

Three factors were probably the source of heterogeneity, and analyzed by meta regression. The LOD and area of DBS were defined as above subgroup analysis. The different PCR methods were defined as real-time PCR, nested-PCR and others (including CMV LC-PCR, conventional PCR combined with nested-PCR, and PCR combined with a hybridization test. The meta regression showed that PCR methods was the factor that influenced the heterogeneity (P = 0.0082).

### Clinical aspects

In total, 5/14 studies [10,13,15,21,23] described the clinical features (symptomatic or asymptomatic) of the enrolled neonates. In asymptomatic infants confirmed as having cCMV infection, the positive rate of DBS PCR assays was 95.90% (117/122), whereas the rate in symptomatic infants was 96.67% (58/60). These values were not significantly different. (P *=* 0.579*,* Fisher’s exact test).

## Discussion

The biggest benefit from screening for cCMV disease is the identification of asymptomatic infants who may develop late onset disease, with symptoms including auditory deterioration. In addition, diagnosing infants with symptomatic infections as early as possible will provide the opportunity to improve the outcomes.

A cost-effective, specific, and sensitive means of newborn screening would make it possible to screen on a large scale. In particular, specimens should be routinely collected from newborns, with CMV DNA kept stable for testing. The test must be amenable to automation at a relatively low cost. More importantly, the test needs to have high sensitivity, especially for neonatal screening. DBS samples are routinely collected for metabolic screening generally within 3 days after birth and PCR assays could be automatically run to test large numbers of specimens.

As shown in Table 3, the length of storage of DBS samples varies from 14 days to 18 years. The length of DBS storage is dependent on the newborn screening policies in different regions. Many states have written policies addressing the storage of DBSs [28]. Therrell et al. [29] surveyed 37 newborn screening programs in the United States with written policies regarding the length of storage, among which the storage time varied from 6 months to 23 years. Johansson et al. [22] has tested 12- to18- year old DBS samples, which was the longest duration of preserving the DBS, and the diagnostic sensitivity and specificity of the test in this study were 81.25% and 100%, respectively. The precision was not affected by the sample storage time. These findings suggest that CMV DNA in DBS samples is stable enough for testing.

Whether cards carrying DBSs with CMV infection will contaminate adjacent cards is another critical problem. In study by Johansson et al. [22], CMV DNA was detected in 6/32 DBS samples stored above or below the CMV positive ones, indicating a transfer of CMV DNA. The possible reason for contamination of adjacent cards may be longtime preservation. However, several studies, such as that by Walter et al., reported no cross-contamination during the period of study [30-33].

Although the pooled sensitivity (84.4%) and pooled specificity (99.9%), and especially the AUC (0.9953) presented better performance for the DBS PCR assays, the moderate heterogeneity (*I*^*2*^ = 45.3%) of the included studies should not be ignored. The subgroup analysis indicated that the study design, LOD, and area of DBS sample all influenced the performance of the DBS PCR assays. The LOD in consideration of the purification is determined by CMV positive specimens in combination with a particular extraction method, followed by a probit analysis. A 95% detection limit denotes that there is a 95% probability that the minimum amount of CMV DNA in whole blood can be detected. In some of the studies, the LOD data was not described. We extracted the information of DNA extraction and PCR methods to calculate the LOD or translate to the number of copies in whole blood (details in Table 1). A higher LOD indicates a lower sensitivity of the PCR assays. The low-LOD subgroup had the higher DOR among all of the groups. In addition, the test performance in large area of DBS group was better than in the other two groups, suggesting that the extraction quality may affect the DBS PCR assays results.

The study design (prospective or retrospective) affected the prevalence of cCMV in the sample groups. In the prospective studies, the prevalence which influences the DOR was always lower than that in the retrospective ones, because more patients were selected in retrospective studies. The results showed that the pooled sensitivity of the prospective studies was 62.3%, which was lower than the 94.5% in retrospective studies. Although the DOR in the prospective studies was higher, “study design” was still tested as a confounder in the meta-regression (data not shown). This results may be explained by patient selection bias due to lack of blinding of the index test operators to the reference test results. For example, 7/8 retrospective studies had a higher risks of patient selection bias. In particular, these studies enrolled infants with CMV infection confirmed by gold standard or with suspected infection and negative controls confirmed not to have infection. The high sensitivity in retrospective studies indicates the test is more suitable for diagnosing cases with risk of long term sequelae and/or following up the children suffered with CMV infection.

We performed a meta-regression to analyze 3 factors to determine the source of heterogeneity. The results showed that the factor of “PCR method” led to inconsistency. The performance of PCR was influenced by several factors, such as DNA extraction, therefore the results indicated that PCR was one of source of heterogeneity. The other two factors were regarded as confounders in the meta-regression analysis.

If the viral load in whole blood cannot reach the detection threshold of the DBS PCR assay, the sensitivity will be affected. The viral load in whole blood from neonates with symptomatic cCMV infection was higher than that in asymptomatic neonates [26,34]. The sensitivity was lower in studies with consecutive cases, which probably enrolled more asymptomatic infants, such as those of Yamamoto et al. [21] and Boppana et al. [2] studies. In the present study, the rates of detection of CMV DNA in DBSs from symptomatic neonates and asymptomatic neonates were similar (95.90% and 96.67%, respectively), based on the data assembled from 5 studies that included information regarding the clinical status of the neonates. However, 2 of the 5 studies [15,23] selected infants with confirmed cCMV infection and performed the test with knowledge of the reference standard’s results, which could inflate the final performance of the test. Therefore, the relationship between the detection rate and clinical features should be explored using a large number of samples, to evaluate the exact value of DBS PCR assays in screening for cCMV infection.

The main limitation of this review is the quality of the included studies. The studies, such as that by Leruez-Ville et al. [12] selected neonates with compatible symptoms, which could have led to an overestimate of the sensitivity and specificity of the test. In addition, the sample sizes in 4 studies were less than 100, which probably resulted in the wide variability in the performance of the tests. Second, the DBSs in the studies were not uniform, including the sample size, different areas of sample, and storage conditions et al. We chose the most important factor, the area of DBS to analyze. The sample sizes may have affected the performance of the screening test, which is of concern given the low prevalence of cCMV 0.42% or 1%, in Canada [35] and Brazil [27], respectively. More studies on DBS assays performed among various prevalence levels of cCMV infection will be needed to evaluate their screening performance.

## Conclusion

Giving sensitivity priority over specificity is particularly useful for epidemiologic surveys of both the prevalence of congenital infection and neonatal screening [23]. The sensitivity of DBS PCR assays may meet the need for retrospective diagnosis, but the diagnostic performance is not robust or sufficient for large-scale universal screening. A DBS sample could be collected and then sent to a laboratory for virology testing in areas that may not have the required equipment.

## Abbreviations

AUC: Area under the ROC curve
cCMV: Congenital cytomegalovirus
CMV: Cytomegalovirus
DBS: Dried blood spot
DOR: Diagnostic odds ratio
LR+: Positive likelihood ratio
LR-: Negative likelihood ratio
NPV: Negative predictive value
PPV: Positive predictive value
QUADAS-2: Quality Assessment of Diagnostic Accuracy Studies 2
SROC: Summary receiver operating characteristic curve
